# Measles Vaccination Coverage and Cases among Vaccinated Persons

**DOI:** 10.3201/eid2108.150284

**Published:** 2015-08

**Authors:** Christian L. Althaus, Marcel Salathé

**Affiliations:** University of Bern, Bern, Switzerland (C.L. Althaus);; Pennsylvania State University, University Park, Pennsylvania, USA (M. Salathé)

**Keywords:** Measles, vaccination, outbreaks, viruses, vaccination coverage, vaccines

**To the Editor:** In December 2014, a measles outbreak that had started at Disneyland Park in Anaheim, California, USA, and subsequently spread to numerous states garnered substantial media attention in the United States. In 2014, the US Centers for Disease Control and Prevention reported the highest number of measles cases (644) since the disease had been declared eliminated from the United States in 2000 (*1*). This number is still relatively lower than the numbers reported from 30 countries of the European Union and the European Economic Area; the highest numbers of measles cases in 2013 were from the Netherlands (2,499 cases), Italy (2,216), the United Kingdom (1,900), and Germany (1,772) (*2*). There is widespread concern that increasing hesitancy to vaccinate in the United States might lead to outbreaks as large as the ones in Europe.

Measles vaccine is highly effective, and analyses of a large measles outbreak at a school in Germany have shown that receipt of >1 doses of vaccine can prevent infection in up to 99% of persons (*3*,*4*). One might therefore be tempted to think that the proportion of measles case-patients who had been vaccinated must be very small. However, when vaccination rates are high, most persons exposed to an infected person will have received >1 doses of vaccine. As a consequence, the expected proportion of persons who had received >1 doses of vaccine among reported measles case-patients will be substantially higher than 1%.

One can derive a simple quantitative relationship between vaccination coverage and the proportion of case-patients who had been vaccinated. Assuming vaccination coverage of *v* and vaccine effectiveness of α, the proportion of the population who are susceptible to measles infection is 1 − α*v*. If all susceptible persons are at the same risk of getting infected, the proportion of vaccinated persons among all case-patients will be *v*(1 − α)/(1 − α*v*). This equation is similar to the screening method that has been used to calculate vaccine effectiveness on the basis of the proportion of case-patients who were vaccinated and vaccination coverage (*5*). Perhaps somewhat counterintuitive at first, the proportion of vaccinated measles case-patients increases with vaccination coverage (Figure).

**Figure:**
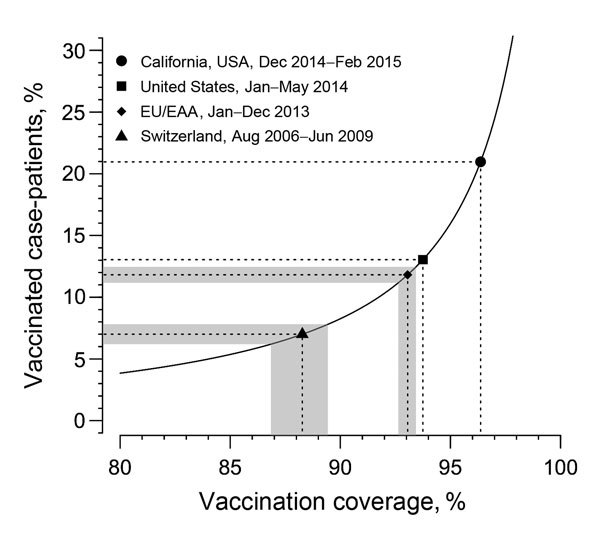
Relationship between vaccination coverage with >1 doses and the proportion of measles case-patients who had been vaccinated. The observed numbers of vaccinated case-patients can be used to infer the vaccination coverage for different populations. Of 62 (21.0%) measles case-patients with known vaccination status in California, USA, 13 had received >1 doses (*6*). Of 230 (13.0%) case-patients with known vaccination status in the United States during January–May 2014, a total of 30 had received >1 doses (*7*). Vaccine effectiveness is assumed to be 99% (*3*,*4*). The shaded areas for the countries of the European Union (EU) and European Economic Area (EEA), and Switzerland correspond to the 95% CIs. 95% CIs are omitted for California and the United States because of the small sample sizes.

We hypothesized that the observed proportion of measles case-patients who had been vaccinated can be used to infer the vaccination coverage in a population at risk (Figure). To this end, we assume a vaccine effectiveness of 99% among persons who had received >1 doses (*3*,*4*). In 2013, countries in the European Union/European Economic Area reported 9,708 measles case-patients for whom vaccination status was known (*2*). Of those, 11.8% had received >1 doses of measles vaccine. On the basis of the relationship derived above, this proportion corresponds to an expected vaccination coverage of 93.1% who had received >1 doses, which is consistent with reported numbers. Switzerland reported 3,850 measles case-patients with known vaccination status from August 2006 through June 2009; of these, 7.0% had been vaccinated with >1 doses (*8*). The inferred vaccination coverage of 88.3% is very close to the reported national level of 87.0% for receipt of >1 doses at 2 years of age (*8*). In contrast, the most recent numbers from the United States suggest that vaccination coverage for receipt of >1 doses is still well over 90%.

Various complexities might affect the relationship between vaccination coverage in a community and the proportion of case-patients who had been vaccinated. First, we assume a vaccine effectiveness of 99% among persons who received >1 doses. Other estimates indicate that vaccine effectiveness is 92% for persons who received 1 dose and 95% for those who received 2 doses (*9*). Assuming that vaccine effectiveness is lower shifts the curve (Figure) to the left and would result in a lower estimate of vaccination coverage. Second, different numbers of persons who received 1 and 2 doses complicate the identification of overall vaccine effectiveness. Third, vaccination status is unknown for some measles case-patients. The proportion of nonvaccinated persons among those case-patients might be higher than that among those known to be vaccinated, also leading to a lower estimate of vaccination coverage. Finally, nonvaccinated persons might be clustered together, and their risk for infection could be higher than that for the general population (*10*). This scenario would imply that the estimated vaccination coverage does not reflect the general population but instead corresponds to a clustered subpopulation among whom vaccination rates are lower. The effects of these complexities warrant further investigation. However, as the examples demonstrate, a model ignoring those effects is in good agreement with empirical data.

Our analysis suggests that the number of vaccinated measles case-patients should be closely followed through surveillance programs. A continuous decrease in the proportion of measles case-patients who had been vaccinated over the years could indicate a decrease in vaccination rates. Conversely, an increase in the proportion of measles case-patients who had been vaccinated would demonstrate the effectiveness of ongoing efforts to increase vaccination rates and could serve as a benchmark toward measles elimination.
